# Nine Myths about Enteral Feeding in Critically Ill Adults: An Expert Perspective

**DOI:** 10.1016/j.advnut.2024.100345

**Published:** 2024-11-17

**Authors:** Tara Ramaswamy, Michael P DeWane, Hassan S Dashti, Meghan Lau, Paul E Wischmeyer, Alexander Nagrebetsky, Jamie Sparling

**Affiliations:** 1 Department of Anesthesia, Critical Care and Pain Medicine, Massachusetts General Hospital, Boston, MA, United States; 2 Department of Surgery, Division of Trauma, Emergency Surgery & Surgical Critical Care, Massachusetts General Hospital, Boston, MA, United States; 3 Department of Anesthesiology, Division of Critical Care Medicine, Duke University Hospital, Durham, NC, United States

**Keywords:** perioperative nutrition, malnutrition, intensive care unit, clinical nutrition, nutrition support, evidence-based practice

## Abstract

Malnutrition is a well-studied and significant prognostic risk factor for morbidity and mortality in critically ill perioperative patients. Common nutrition myths in the critically ill may prevent early, consistent, and adequate delivery of enteral nutrition. We outlined 9 common intensive care unit (ICU) nutrition misconceptions and our recommendations to optimize enteral nutrition in critically ill patients based on the review of available literature. Our approach is to treat every patient admitted to the ICU as at risk for malnutrition and to initiate enteral nutrition upon admission in the absence of contraindications. Early enteral nutrition via the gastric route is more beneficial than delaying feeding while awaiting small bowel access and daytime-intermittent nutrition support can safely be initiated over continuous feeding. Gastric residual volumes to assess feeding tolerance should no longer be routinely measured. For perioperative nutrition, we recommend continuing enteral nutrition for most patients with secure airways undergoing anesthesia and resuming nutrition within 24 h of abdominal surgery; even patients with open abdomens can be safely fed in the absence of bowel injury. Critically ill patients who are proned, paralyzed, and on vasopressors can usually continue enteral nutrition. Finally, continuing enteral nutrition before extubation may optimize nutrition without compromising extubation success. In this review, we highlight several common misconceptions regarding ICU nutrition that may prevent achieving nutrition goals and subsequently lead to increased malnutrition, morbidity, and mortality. This knowledge may contribute to increased implementation of early and consistent enteral nutrition strategies to improve outcomes in critically ill adult patients.

**Statement of Significance**

There are several common misconceptions regarding intensive care unit (ICU) nutrition that may exacerbate malnutrition. We describe 9 common ICU nutrition misconceptions and recommendations to optimize nutrition in critically ill patients based on the review of available literature.

## Introduction

**FIGURE 1:**
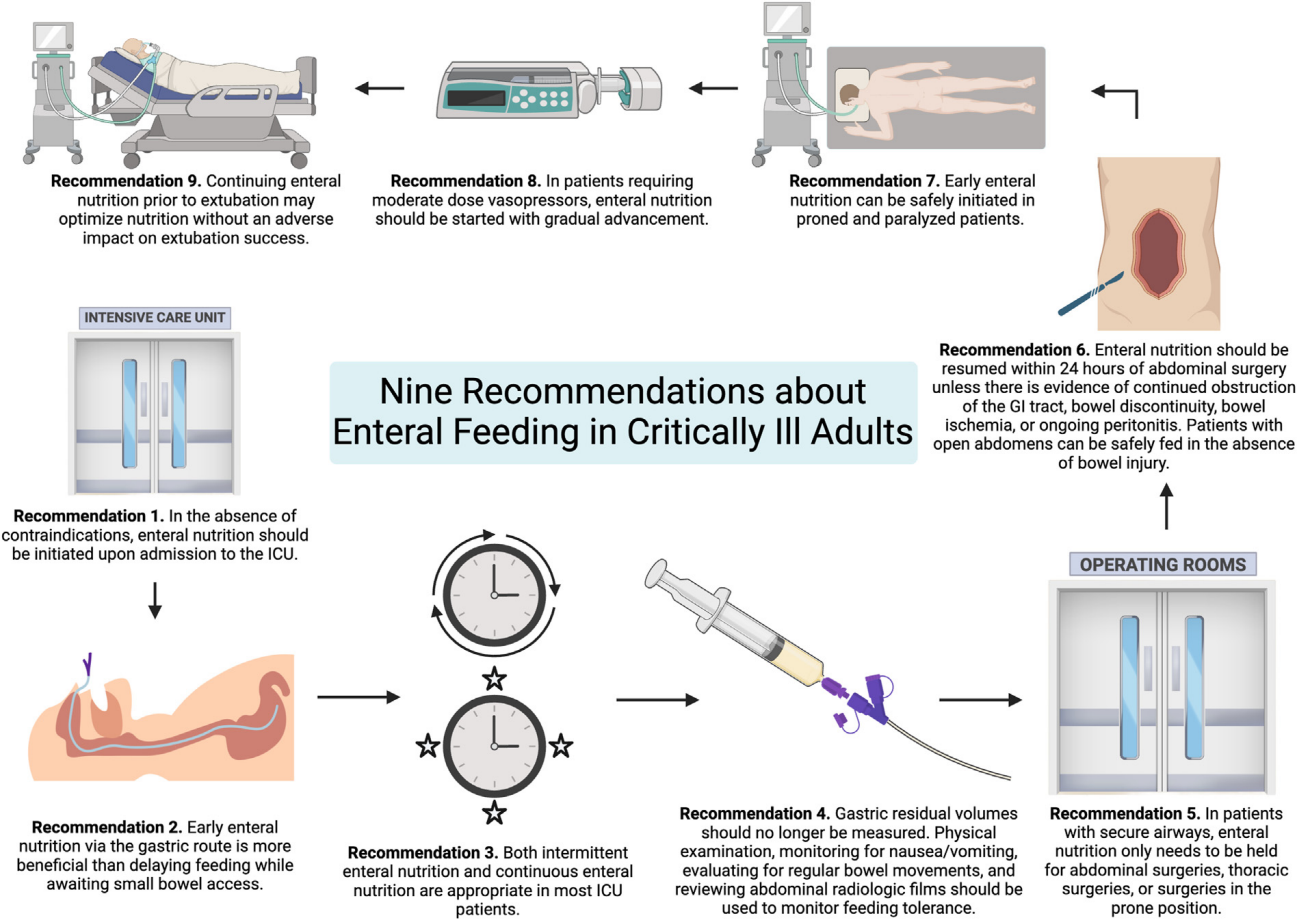
Summary of 9 common intensive care unit (ICU) nutrition misconceptions.

Malnutrition is a well-studied and significant prognostic risk factor for morbidity, mortality, and length of stay in critically ill patients [[1], [2], [3], [4], [5]]. Adverse outcomes related to malnutrition include increased infection rates, longer length of stay, higher readmission rates, muscle wasting, pressure ulcers, functional loss with increased fall risk, higher treatment costs, and increased mortality [2,6]. The American Society of Parental and Enteral Nutrition (ASPEN) and European Society for Clinical Nutrition and Metabolism (ESPEN) have recently updated recommendations and guidelines for enteral and parental nutrition in critically ill adult patients [7,8]. Despite these recommendations, iatrogenic malnutrition remains common, with the prevalence of malnutrition in intensive care unit (ICU) patients ranging between 38% and 78% [1,5]. More recently, 92.2% [95% confidence interval (CI): 85.9%, 96.8%] of critically ill patients with COVID-19 were at risk for malnutrition [9]. In an international multicentre observational study, only ∼60% of prescribed calories and proteins were delivered to adult ICU patients over the first 12 d of ICU stay, and the mean delay in initiation of enteral nutrition was 46.5 h (range of site averages: 8.2–149.1 h) [10]. This inadequate intake commonly persists for 1–2 wk or greater after ICU admission [1]. Lack of adherence to guidelines may be perpetuated in part by common nutrition myths in the ICU that prevent early, consistent, and adequate delivery of nutrition in patients. Indeed, a 2021 study of ICU physicians and nurses demonstrated that lack of familiarity and understanding of both society and local guidelines, as well as inadequate evidence, are barriers to adoption of effective enteral nutrition in the ICU [11]. We have outlined 9 common misconceptions below and our clinical approach to best optimize enteral nutrition in critically ill adult patients (Figure 1, Table 1). The focus of this review is on enteral nutrition in adult critically ill patients, and our recommendations do not apply to the critically ill pediatric population.

**TABLE 1:** Nine common intensive care unit (ICU) enteral nutrition misconceptions and corresponding recommendations.

Myth	Recommendation
1. Early initiation of enteral nutrition is not necessary.	In the absence of contraindications, enteral nutrition should be initiated upon admission to the ICU.
2. Significant clinical differences exist between prepyloric and postpyloric feeding tubes.	Early enteral nutrition via the gastric route is more beneficial than delaying feeding while awaiting small bowel access.
3. Continuous is preferred over bolus enteral nutrition for the prevention of aspiration and intolerance.	Both intermittent enteral nutrition and continuous enteral nutrition are appropriate in most ICU patients.
4. High gastric residual volumes lead to increased risk of aspiration.	Gastric residual volumes should no longer be measured. Physical examination, monitoring for nausea/vomiting, evaluating for regular bowel movements, and reviewing abdominal radiologic films should be used to monitor feeding tolerance.
5. Enteral nutrition needs to be held for patients with a secure airway undergoing anesthesia.	In patients with secure airways, enteral nutrition only needs to be held for abdominal surgeries, thoracic surgeries, or surgeries in the prone position.
6. Enteral nutrition should be delayed after abdominal surgeries.	Enteral nutrition should be resumed within 24 h of abdominal surgery unless there is evidence of continued obstruction of the GI tract, bowel discontinuity, bowel ischemia, or ongoing peritonitis. Patients with open abdomens can be safely fed in the absence of bowel injury.
7. Proned and paralyzed patients should be NPO.	Early enteral nutrition can be safely initiated in proned and paralyzed patients.
8. Enteral feeds should be stopped in patients on vasopressors.	In patients requiring moderate-dose vasopressors, enteral nutrition should be started with gradual advancement.
9. Enteral nutrition should be held before extubation.	Continuing enteral nutrition before extubation may optimize nutrition without an adverse impact on extubation success.

Abbreviations: GI, gastrointestinal; NPO, nil per os.

Malnutrition in the ICU is nuanced, and patients can be difficult to evaluate. Massive fluid resuscitation, daily diuresis, and rapid wasting of lean muscle can make it challenging to assess weight changes. BMI may be within a normal range despite malnutrition and significant loss of lean body mass. Malnutrition is much less apparent when the ICU patient has obesity, and these patients represent a high-risk population prone to sarcopenia and malnutrition. Indeed, 57% of hospitalized patients with a BMI >25 kg/m^2^ show clinical evidence of malnutrition [12]. Commonly utilized nutritional markers such as albumin and prealbumin are inadequate markers of nutrition because of their status as negative acute phase proteins whose low values reflect a response to inflammation and hydration status [13,14]. Nutritional scoring systems include the Nutrition Risk Score (2002), the Nutrition Risk in the Critically Ill Score, the Subjective Global Assessment, the Malnutrition Universal Screening Tool, and the Global Leadership Initiative on Malnutrition (GLIM) criteria. These systems integrate medical history, disease severity, physical examination, and markers of malnutrition including weight loss and appetite changes. The GLIM assessment, in particular, can be repeated on discharge to assess the development of malnutrition during a patient’s ICU stay. However, many of these parameters are often difficult to measure in ICU patients and thus clinicians must apply these tools in the context of the larger clinical picture.

Given the difficulty in assessing nutritional status and the high propensity for underfeeding in the ICU, our approach is to treat every patient in the ICU as at risk for malnutrition and requiring prompt initiation of enteral nutrition unless contraindicated.

## Discussion

### Myth 1: early initiation of enteral nutrition is not necessary

Early enteral nutrition is beneficial, consistently recommended by clinical guidelines and shown to improve outcomes [15]. Unfortunately, the average initiation time for enteral nutrition in ICU patients globally remains 46.5 h after ICU admission [10]. Because of provider and nursing concerns regarding tolerability of enteral nutrition, the decision to initiate enteral nutrition is often delayed. Clinical studies have shown that initiation delay prevents achieving calorie and protein goals and subsequently contributes to adverse effects on recovery [[16], [17], [18]], including poor prognosis and increased mortality [19,20]. Furthermore, initiation of enteral nutrition upon admission is associated with reduced length of ICU stay and ventilator time compared with the initiation of enteral nutrition at 24–48 h after admission [16]. Further data demonstrate that early enteral nutrition compared with late enteral nutrition is associated with reduced infectious morbidity in ICU patients [[21], [22], [23]]. Recent ASPEN and ESPEN guidelines recommend commencement of enteral nutrition within 24–48 h of critical illness in ICU patients, with moderate caloric (12–25 kcal/kg/d) and protein (1.2–2.0 g/kg/d) targets [7,8,24]. These recommendations are [7,8,24] consistent with the results of 3 meta-analyses comparing early with late enteral nutrition concluding that early initiation is associated with statistically significant reductions in infections, hospital length of stay, pneumonia rates, and mortality [[25], [26], [27]]. Although the French-Speaking ICU Nutritional Survey (FRANS) prospective cohort study showed an association with higher 28-d mortality in patients receiving early enteral nutrition, this study was initiated nearly a decade ago (2015) and reflects earlier guidelines with higher energy targets [28,29]. Several studies, including the recent multicentre randomized controlled trial, NUTRIREA-3 (Low versus standard calorie and protein feeding in ventilated adults with shock), have demonstrated no difference in 90-d mortality (absolute difference 1.5%, 95% CI: –5.0, 2.0; *P* = 0.41) with faster recovery times as measured by readiness for ICU discharge [8.0 d compared with 9.0 d, hazard ratio (HR): 1.12, 95% CI: 1.02, 1.22, *P* = 0.015] and lower rates of vomiting (HR: 0.77, 95% CI: 0.67, 0.89; *P* < 0.001), diarrhea (HR: 0.83, 95% CI: 0.73, 0.94; *P* = 0.04), and bowel ischemia (HR: 0.50, 95% CI: 0.26, 0.95; *P* = 0.030) among patients receiving early, low-calorie, and low-protein targets compared with early, standard calorie and protein targets [30]. Similarly, in the EFFORT Protein trial (The effect of higher protein dosing in critically ill patients), a randomized controlled trial across 85 ICUs in 16 countries, delivery of higher doses of protein (≥2.2 g/kg/d) to intubated patients at high nutritional risk, compared with lower doses of protein (≤1.2 g/kg/d), did not improve the rate of discharge alive from the hospital (46.1% compared with 50.2%, 95% CI: 0.77, 1.07; *P* = 0.27) with worsened outcomes among patients with acute kidney injury and higher baseline organ failure scores receiving higher protein targets [31]. The results of these trials are consistent with the distinct metabolic phases during acute illness—an early acute phase characterized by hemodynamic instability and insulin resistance, wherein lower to moderate protein and calorie targets are beneficial and overfeeding may be detrimental because of endogenous substrate production (ICU days 1 and 2), a late acute phase (ICU days 3–7), and a recovery phase (after ICU day 7) [15]. Thus, in the absence of contraindications, enteral nutrition with low-to-moderate calorie and protein targets should be initiated upon admission to the ICU.

### Myth 2: significant clinical differences exist between prepyloric and postpyloric feeding tubes

For most patients, including those without a mechanical bowel or gastric outlet obstruction, there is no significant difference between prepyloric or postpyloric tube placement. Gastric tubes are usually easier to place and performed more quickly. In a large multicentre, prospective, randomized controlled trial comparing gastric with small bowel enteral nutrition in critically ill patients with elevated gastric residual volumes (GRVs) within 72 h of ICU admission, Davies et al. [32] found no difference in clinical outcomes between groups, including in-hospital mortality (13% compared with 14%, *P* = 0.88), nutrient delivery (71% compared with 72% estimated energy requirements, *P* = 0.66), and risk of aspiration (4% compared with 5%, *P* = 76). Additional randomized trials have shown that although postpyloric feeding may reduce the incidence of pneumonia, it has no impact on mortality (13% compared with 14%, *P* = 0.88), nutrient delivery (71% compared with 72% estimated energy requirements, *P* = 0.66), or risk of aspiration (4% compared with 5%, *P* = 0.76) [[32], [33], [34]]. Per ASPEN guidelines, if timely obtainment of a small bowel enteral access device is not feasible, early enteral nutrition via the gastric route may be more beneficial than delaying feeding initiation while awaiting small bowel access [8]. In our opinion, postpyloric enteric access should be more strongly considered in patients with delayed gastric emptying after foregut surgery such as gastrectomy, distal pancreatectomy, gastrojejunal bypass, and others.

### Myth 3: continuous is preferred over bolus enteral nutrition for the prevention of aspiration and intolerance

In most ICUs, enteral nutrition is provided in a continuous manner rather than in a “bolus” or intermittent method of administration. This practice is ostensibly to limit gastrointestinal intolerance and glucose variability [35]. However, ESPEN guidelines indicate that there is little evidence that continuous feeding is safer than bolus infusions [7] and a recent meta-analysis suggests that the continuous infusion of nutrition is associated with an increased risk of constipation [relative risk (RR): 2.24, 95% CI: 1.01, 4.97] and no difference in mortality (RR: 0.71, 95% CI: 0.40, 1.38), pathogenic bacterial colonization in the oropharynx or upper gastrointestinal tract (RR: 1.08, 95% CI: 0.87, 1.34), pneumonia (RR: 1.08, 95% CI: 0.45, 2.59), diarrhea (RR: 0.62, 95% CI: 0.35, 1.08), or elevated GRV (RR: 1.16, 95% CI: 0.65, 2.05) [36]. Bolus feeding is more physiologic and may provide several advantages such as limiting interruptions, increasing cost effectiveness, maintaining muscle mass, and improving patient mobility, and has been demonstrated to be safe for jejunostomy feeds as well as gastric feeds [35,37]. An evolving field of chrononutrition supports aligning nutrition with the biological clock, and evidence from healthy adults suggests that feeding at night results in circadian misalignment, a risk factor for cardiometabolic disease [38]. Future studies are needed in critically ill patients, who have multiple causes of altered sleep–wake cycles. Our recommendation is that both intermittent enteral nutrition and continuous enteral nutrition are appropriate modes of administration in ICU patients.

### Myth 4: high GRVs lead to increased risk of aspiration

GRV is the amount aspirated from the stomach after the administration of enteral feeds and is an indicator of a functioning gastrointestinal tract. A common practice in ICU nutrition therapy is to frequently measure GRV and subsequently pause enteral feeds if residual volumes are elevated, with the goal of reducing the risk of aspiration. Elevated GRV is a common reason for interrupting enteral nutrition preventing the goal of enteral feeding rates to be reached, and there is currently little evidence suggesting that GRV measurement is effective in reducing the risk of aspiration or other pulmonary complications [[39], [40], [41], [42]].

A large randomized controlled trial in mechanically ventilated patients in the ICU found no relationship between GRV monitoring and ICU complications, including risk for aspiration or days on mechanical ventilation [43]. Patients randomly assigned to not having GRV monitored received a higher percentage of goal calories. Another multicentre randomized controlled trial found that there were no important differences in using a GRV limit of 500 mL compared with 200 mL in terms of meeting nutrition goals and incidence of gastrointestinal (GI) complications, ICU-acquired pneumonia, days on mechanical ventilation, and ICU length of stay [44]. Furthermore, the most recent ASPEN/Society of Critical Care Medicine Critical Care Guidelines published in 2016 recommended against using GRVs as part of routine care to monitor ICU patients receiving enteral nutrition, and for ICUs where GRVs are still measured, holding enteral nutrition for GRVs <500 mL in the absence of other signs of intolerance should be avoided [8]. However, many ICUs still use elevated GRV as a criterion to hold enteral nutrition, and there is wide variation in practice, with many providers holding enteral nutrition at lower GRVs.

Although symptoms may be difficult to assess in intubated patients, several alternative strategies may be used to monitor enteral feeding tolerance in critically ill patients: performing regular physical examinations, monitoring for nausea/vomiting, evaluating for the presence of flatus and for regular bowel movements, and reviewing abdominal radiologic films [45].

### Myth 5: enteral nutrition needs to be held for patients with a secure airway undergoing anesthesia

As stated previously, fewer than half of patients reach their energy and protein goals during their ICU stay. Fasting after midnight for diagnostic tests and procedures affects approximately one-third of ICU patients and is associated with ≤25% of cessation time [[46], [47], [48], [49]]. There are currently no clear clinical guidelines regarding preprocedural discontinuation of enteral nutrition in patients with secure airways, and practices vary widely across different institutions [50]. Given that 20% of scheduled procedures are delayed until the following day, extended fasting inflicted by interruptions in enteral nutrition can result in clinically significant nutritional deficits and prolonged ICU and hospital stay [51]. Such prolonged periods of fasting may worsen ileus [52]. In one study, patients undergoing frequent surgical procedures randomly assigned to continue enteral nutrition throughout the entirety of their perioperative and intraoperative courses had significantly fewer infections than those patients for whom enteral nutrition was stopped for each procedure [53]. Our current practice is that in patients with secure airways, enteral nutrition only needs to be held for abdominal surgeries, thoracic surgeries, or surgeries in the prone (or lateral decubitus depending on institution preference) position. Continuation of enteral nutrition intraoperatively may be considered for patients without risk factors for aspiration and for whom the risk of fasting is heightened, such as patients with severe burn injury requiring serial debridements [51].

### Myth 6: enteral nutrition should be delayed after abdominal surgeries

Feeding within 24 h after abdominal surgery is critical in reducing postoperative ileus, bowel wall edema, and in reducing complications and mortality [8]. In most patients undergoing abdominal surgeries, enteral nutrition initiation within 24–48 h is recommended. Even in a patient with fresh anastomosis, it is not necessary to withhold enteral nutrition or administer clear liquids only [7,8]. When feasible, enteral nutrition is always prioritized over parenteral nutrition [8]; a meta-analysis of 13 trials with over 1000 patients showed that absolute mortality was reduced from 6.8% to 2.4% with use of early enteral nutrition postoperatively compared with standard therapy with no increase in aspiration [54]. In a subsequent systematic review and meta-analysis, there was no worsening effect on anastomotic dehiscence in early enteral nutrition [55,56]. Enteral nutrition should only be held postoperatively if there is evidence of continued obstruction of the GI tract, bowel discontinuity, bowel ischemia, or ongoing peritonitis. Even in patients with persistent anastomotic leak or with internal or external fistulas, access to the distal part of the gut can be used [8]. Advancing patients to clear liquids after surgery before solid foods does not decrease intolerance or postoperative complications and may result in increased aspiration [[57], [58], [59]].

When an “open abdomen” is necessitated because of the inability to close the abdominal cavity without excessive intraabdominal pressure or because of ongoing intraabdominal pathology, providers may be reluctant to initiate enteral nutrition in patients. However, retrospective data suggest that initiation of enteral nutrition in patients with an open abdomen is safe and associated with significant reductions in intraabdominal complications and time to abdominal fascial closure, as well as mortality [[60], [61], [62]]. In the absence of bowel injury, early enteral nutrition is recommended in patients with an open abdomen [8].

### Myth 7: proned and paralyzed patients should be fasted

Prone positioning has been shown to reduce mortality in acute respiratory distress syndrome [63] and its adoption has increased during the COVID-19 pandemic [64]. The available literature on the effect of enteral nutrition administered in the prone position is scarce and of varying quality, as earlier studies often used GRV as a surrogate for gastrointestinal tolerance [65]. There are mixed results with regard to the effects of prone positioning on increasing GRVs and vomiting, but no evidence on increasing risk of pneumonia or death [65]. In a systematic review, enteral nutrition while prone is feasible and well tolerated while allowing achievement of nutritional goals [66]. Per updated ASPEN guidelines for COVID-19: “Retrospective and small prospective trials show enteral nutrition in prone patients is not associated with increased risk of GI or pulmonary complications, thus we recommend early EN in prone patients” [67].

Neuromuscular blockade, often employed to assist ventilator synchrony and reduce skeletal muscle oxygen consumption in intubated patients, acts on skeletal muscle through nicotinic acetylcholine receptors at neuromuscular junctions. Neuromuscular blockers have no effect on smooth muscle, the type of muscle found in the gut. Previous data have shown that neuromuscular blockade does not affect gastric peristalsis [68], and enteral nutrition in paralyzed patients may reduce mortality, length of stay, and hospital-acquired pneumonia [69]. Enteral nutrition can safely be delivered in patients paralyzed with neuromuscular blockade.

### Myth 8: enteral feeds should be stopped in patients on vasopressors

Often concerns are raised that enteral nutrition can worsen ischemia and reperfusion injuries in patients with sepsis, hypotension, and presumed GI dysmotility, leading to patients not receiving nutrition until vasopressors are weaned off. However, several studies support enteral nutrition within 48 h of ICU admission in patients requiring small or moderate doses of vasopressors [8,23,70]. In the NUTRIREA-2 study, over 2000 patients with a median vasopressor requirement of 0.5 μg/kg/min norepinephrine were randomly assigned to receive full enteral nutrition or parenteral nutrition. There was an absolute risk of 2% of bowel ischemia in patients receiving enteral nutrition, which was a statistically significant increase from the parenteral group (risk of <1%) [71]. However, the patients in the intervention group were on massive doses of vasopressors (median 45 μg/min norepinephrine) and receiving full enteral nutrition. In addition, there were no differences in 28-d mortality, the study’s primary endpoint. Additional aggregated data suggest that enteral nutrition might decrease infectious complications from vasopressor use via the protection of gastrointestinal wall integrity [22,72]. In a large health outcome study, 28-d mortality was significantly lower in the early enteral nutrition group in low-dose and medium-dose norepinephrine groups with no signal of risk for adverse outcomes in the high-dose norepinephrine group [73]. Therefore, in patients requiring stable low-to-moderate dose vasopressors (<0.3 μg/kg/min), enteral nutrition should be started with gradual advancement as the patient tolerates [74]. Patients should initially receive gastric feeds rather than postpyloric feeds and clinicians should monitor for signs and symptoms of intolerance including distension, nausea, vomiting, abdominal pain, and unexplained lactate elevation [70]. In cases of uncontrolled shock, enteral feeding should not be initiated until the patient is stable and improving from a shock perspective, based on trends in markers of end-organ perfusion (for example, lactate) and vasopressor requirements.

### Myth 9: enteral nutrition should be held before extubation

A common practice is to hold enteral feeds at midnight before a morning spontaneous breathing trial or at a set time interval before planned extubation; however, this can result in prolonged fasting times if patients are not extubated early the next morning. Fasting periods are further prolonged when clinicians keep recently extubated patients nil per os in the case of reintubation. Although ASPEN guidelines recommend minimizing fasting duration, there are currently no explicit guidelines on whether enteral nutrition should be paused before extubation. A recent multicentre randomized controlled trial of 1130 patients compared continued enteral nutrition until extubation with a 6-h fasting period with gastric suctioning before extubation. There were no differences in extubation failure between the groups, and the rate of pneumonia within 14 d was lower in the group assigned to continued enteral nutrition [75]. Therefore, continuing enteral nutrition before extubation may optimize nutrition while minimizing risks of underfeeding. Before extubation, the stomach should be suctioned empty, if possible.

## Conclusion

In this review, we highlight several common misconceptions regarding ICU nutrition that may prevent achieving nutrition goals and subsequently lead to increased malnutrition, morbidity, and mortality (summarized in Figure 1 and Table 1). We hope that this work contributes to increased implementation of early and consistent nutrition strategies to improve outcomes in our critically ill adult patients.

## Author contributions

The authors’ responsibilities were as follows – TR: guarantor of the content of the manuscript and responsible for the writing and final content; JS: responsible for the design of the manuscript, and significantly contributed to the writing of the manuscript; MPD, HSD, PEW, AN: contributed to the design and writing of the manuscript; ML: contributed to the figure creation; and all authors: read and approved the manuscript in its final version.

## Funding

This review was not supported by a funding source.

## Conflict of interest

HSD is an Editor for Advances in Nutrition and played no role in the journal’s evaluation of the manuscript. The other authors declared no potential conflicts of interest with respect to the research, authorship, or publication of this article.
